# Management and Treatment of an Emergent Nonpuerperal Uterine Inversion With a Leiomyoma

**DOI:** 10.7759/cureus.90180

**Published:** 2025-08-15

**Authors:** Michael J Padron, Ishaan Dutta, Daphne Bazile

**Affiliations:** 1 Obstetrics and Gynecology, Liberty University College of Osteopathic Medicine, Lynchburg, USA; 2 Obstetrics and Gynecology, Bon Secours - Southside Medical Center, Petersburg, USA

**Keywords:** abdominal hysterectomy, fibroid, leiomyoma, nonpuerperal uterine inversion, uterine inversion, vaginal myomectomy

## Abstract

Uterine inversion is a rare medical emergency that occurs when the uterus inverts and, at times, protrudes from the vagina. Uterine inversions can be puerperal, related to childbirth, or nonpuerperal, often secondary to tumors within the uterine cavity. Our case presents a nonpuerperal uterine inversion caused by a pedunculated submucosal leiomyoma. The case follows a patient from her initial presentation through the resolution of her condition with a vaginal myomectomy and abdominal hysterectomy.

## Introduction

Uterine fibroids, or leiomyomas, are common benign smooth muscle tumors of the uterus, frequently affecting women of reproductive age. These fibroids are categorized based on their location within the uterine wall. Submucosal fibroids are located just beneath the endometrial lining and can protrude into the uterine cavity, causing heavy menstrual bleeding and reproductive complications. Intramural fibroids grow within the uterine muscle, often leading to pelvic pain, pressure, and abnormal bleeding. Subserosal fibroids develop on the outer surface of the uterus and can exert pressure on nearby organs, leading to discomfort and bulk symptoms [1].

While fibroids are usually benign, they can sometimes contribute to more serious gynecological emergencies such as uterine inversion. Uterine inversion typically occurs when the uterus folds in on itself and protrudes through the cervix. This medical emergency is commonly observed postpartum secondary to traction from the umbilical cord. However, in rare cases, such as our patient, nonpuerperal uterine inversion can occur when underlying pathologies, such as large submucosal fibroids, large endometrial polyps, or other intrauterine masses, exert sustained traction on the uterine fundus. This pressure, especially in the presence of a thinned or weakened uterine wall, leads to gradual invagination of the fundus through the cervical canal. As the fibroid descends, it pulls the uterus with it, resulting in inversion [2].

Anatomical complications of nonpuerperal uterine inversion include damage to the pelvic structures, disruption of the uterine vasculature, and tearing of the supportive ligaments [3]. If not promptly addressed, it can lead to ischemia and necrosis of the uterus, posing life-threatening risks. The management of nonpuerperal uterine inversion often requires immediate surgical intervention to restore the uterus to its normal position and manage any underlying pathology such as the removal of fibroids [4].

## Case presentation

A 34-year-old female with multiple medical conditions, including an unspecified developmental delay, uterine leiomyomas, and a suspected uterine prolapse per prior visit, presented to the emergency department for the evaluation of a mass extending from her vagina. The patient’s mother reported noticing the mass earlier in the day, after the patient used the restroom. Prior to this, prior pelvic examinations were unable to be tolerated due to the patient’s baseline. Per the patient’s caregiver, the patient had previously experienced abnormal menstrual bleeding. When evaluated by her primary care provider for this, the patient was placed on hormonal therapy, which resulted in minimal improvement of her symptoms. A pelvic examination under anesthesia had been scheduled but was canceled by the patient’s caregiver.

**Figure 1:**
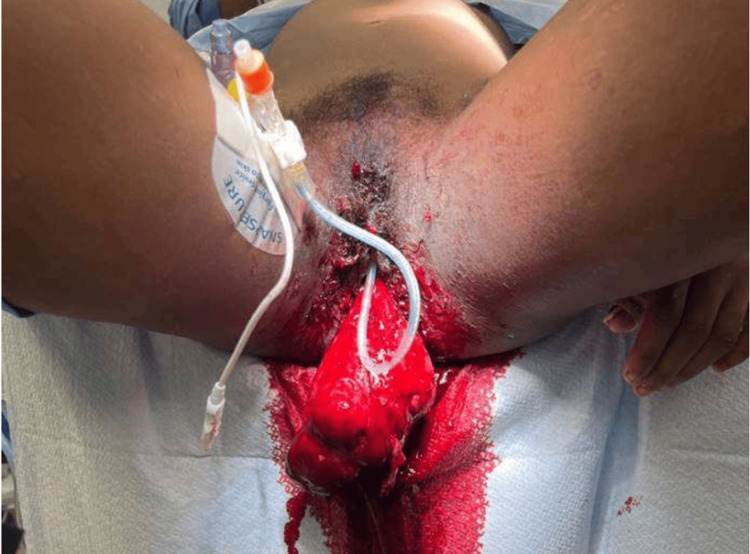
Uterine inversion with a pedunculated leiomyoma

Upon entering the emergency department, the patient was found to have a blood pressure of 154/112 mmHg, a pulse of 126 bpm, and a temperature of 98.6 °F. Physical examination findings included a large pedunculated mass protruding from the patient’s vagina (Figure 1). The patient’s CBC included a hemoglobin of 11 g/dL and a hematocrit of 37%. Within 30 minutes of arrival, the patient’s blood pressure had dropped to 77/55 mmHg with a heart rate of 105 bpm. The patient was then emergently taken to the operating room for further management and prevention of blood loss.

**Figure 2:**
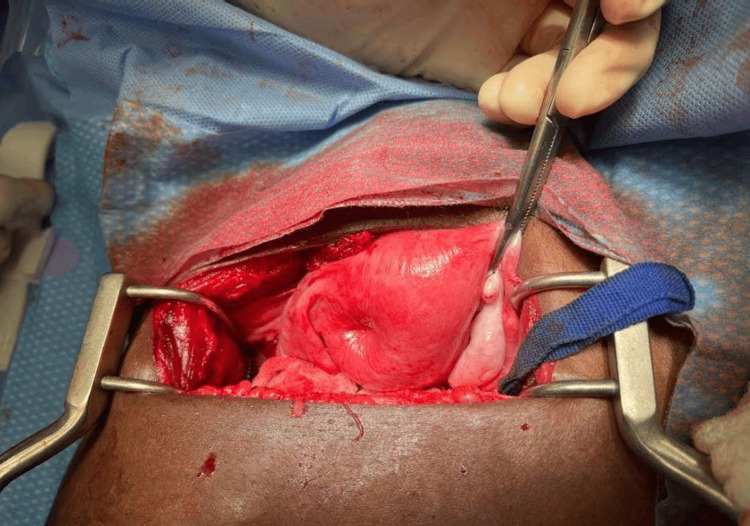
Uterus inside the pelvis post vaginal myomectomy

An exploratory abdominal laparotomy was performed first, which revealed an inverted uterus. It was deemed that the uterus, and specifically a pedunculated leiomyoma, had protruded through her vagina. The leiomyoma was then excised at the base of the stalk, enabling the uterus to return to her pelvis (Figure 2). Due to continued hemorrhage, an open abdominal hysterectomy without salpingo-oophorectomy was performed without major complications.

The leiomyoma and the uterus were sent to pathology. The pathology report read: a 220 g ragged pink-tan nodular structure measuring 7.1 cm, which was focally smooth and hemorrhagic, consistent with possible uterine serosa. The specimen was serially sectioned to reveal a pink to pale-tan, whorled cut surface. The uterine serosa is pink-tan and smooth on the posterior surface with a focally disrupted anterior surface. The cervix is pink-tan, focally hyperemic, and unremarkable.

The patient was then admitted to the hospital for further observation. She tolerated the procedure well and was discharged a couple of days later.

## Discussion

Given the patient’s change in blood pressure and physical exam findings, the decision to bring the patient to the operating room was made prior to a further workup and evaluation to prevent further blood loss. Traditionally, from a surgical standpoint, there is an attempt to revert the uterus to the pelvis. This is a primary objective because repositioning the uterus can prevent further hemorrhage. Several techniques can be utilized in this situation to revert the uterus, including the Haultain procedure [3] and the Johnson Method [5]. In our case, the Johnson method, which includes gently pushing the fundus back through the cervix for repositioning [5], was attempted but was unsuccessful. The physician decided that it would be best to perform a vaginal myomectomy to stop the hemorrhage. Following excision of the pedunculated submucosal leiomyoma, the uterus was able to return to the pelvis. An abdominal hysterectomy was then performed on this patient to stabilize her condition due to continued hemorrhage.

Studies estimate that 69.5% of nonpuerperal uterine inversions are caused by leiomyomas. From a review of 133 surgical cases, only 27% were handled with a combined abdominal and vaginal procedure. Yet, what makes this case even more rare was the vaginal myomectomy performed before repositioning of the uterus. According to the literature, only 6.8% of cases had repositioning of the uterus following surgical resection of the tumor. These statistics highlight the rarity of this case and, more specifically, the management decision made to successfully handle the situation [6].

Often, in situations of uterine inversions, decisions made to perform a hysterectomy are dependent on the patient’s fertility plans [3]. In our case, the patient’s guardian stated prior to the procedure that the patient is not sexually active and there is no desire to preserve her fertility. Therefore, prior to the procedure, her guardian approved the hysterectomy as it would aid in stopping the hemorrhage. 

An unfortunate reality that has played a role in this case was the patient’s developmental disability, limiting prior examinations. It is documented in her chart that the decision was made by her Obstetrician and Gynecologist to perform a pelvic examination of the patient under anesthesia, as the patient had been combative during previous examinations. However, when the examination and procedure were scheduled, the patient was not brought to the appointment by her guardian. The Obstetrician and Gynecologist had also been concerned about the patient’s abnormal bleeding; therefore, they ordered an ultrasound of the pelvis. Multiple transvaginal ultrasounds could not be performed. The patient had presented to the emergency department on a separate occasion and had undergone a transabdominal ultrasound of the pelvis. This ultrasound was conducted three and a half months prior and found two uterine leiomyomas, measuring 5.2 cm and 5.1 cm. The radiology report also indicated that the study was limited by patient cooperation. Given these ultrasound findings, there is a possibility that if the patient had been brought to the prior examination under anesthesia, the physician would have been able to identify the inversion sooner and prevent this emergent situation.

Future management strategies for similar cases should involve earlier sedation-assisted examinations or imaging techniques to assess intrauterine pathology before an acute emergency arises. Education and counseling of caregivers regarding the potential risks of untreated fibroids, including uterine inversion, are also essential in preventing recurrence and ensuring timely intervention.

## Conclusions

Nonpuerperal uterine inversion is both a rare and emergent condition often caused by a leiomyoma. Management of this condition is variable, but the end goal of hemodynamic stability remains the same. Cases such as the one presented are important to study to understand the medical decision-making that is required to successfully treat this condition.
